# Misconceptions and stigma against people living with HIV/AIDS: a cross-sectional study from the 2017 Indonesia Demographic and Health Survey

**DOI:** 10.4178/epih.e2021094

**Published:** 2021-11-06

**Authors:** Desi Suantari

**Affiliations:** Faculty of Sciences and Technology, Universitas Islam Negeri (UIN) Sunan Kalijaga Yogyakarta, Sleman, Indonesia

**Keywords:** Human immunodeficiency virus, Education, Knowledge, Indonesia

## Abstract

**OBJECTIVES:**

Data are not available in Indonesia to measure the main indicators of zero new infections, zero acquired immune deficiency syndrome (AIDS)-related deaths and zero discrimination. This study aimed to determine factors related to misconceptions about human immunodeficiency virus (HIV) transmission and the stigma against people living with HIV/AIDS (PLWHA) in Indonesia

**METHODS:**

This cross-sectional study used secondary data from the 2017 Indonesia Demographic and Health Survey (IDHS). The sample was women and men aged 17–45 years and married (n=3,023).

**RESULTS:**

Education and wealth index quintile were significantly related to misconceptions about HIV transmission. Respondents with low levels of education were more likely to have misconceptions about HIV transmission. Respondents who were in the poorest, poorer, middle, and richer quintiles of the wealth index were more likely to have misconceptions about HIV transmission than those in the richest quintile. Educational level, employment status, and wealth index quintile were predictors of stigma against PLWHA.

**CONCLUSIONS:**

There are still many Indonesian people with misconceptions about HIV transmission and stigma against PLWHA. Future studies should focus on educational programs or interventions aimed at increasing public knowledge and awareness, promoting compassion towards PLWHA, and emphasizing respect for the rights of PLWHA. These interventions are particularly important for populations who are uneducated and living in poverty.

## INTRODUCTION

Human immunodeficiency virus (HIV) is a virus that infects white blood cells, weakening the human immune system, while acquired immune deficiency syndrome (AIDS) is a set of disease symptoms that arise in the later stages of immune deficiency caused by HIV infection [1]. The virus can be transmitted by sexual contact, sharing needles to inject drugs, and from mother to baby during pregnancy, birth, or breastfeeding. The virus is not transmitted by air or water, saliva, sweat, tears, closed-mouth kissing, insects or pets, or sharing toilets, food, or drinks [2].

Globally, in 2020, it was estimated that 37.7 million (30.2 to 45.1 million) people were living with HIV. In Southeast Asia, there were 3.7 million people living with HIV, 100,000 (71.000 to 130.000) people newly infected with HIV, and 82,000 (55,000 to 130,000) people dying from HIV-related causes [3]. Although data on HIV/AIDS fluctuate, cases in Indonesia continue to increase from year to year. Over the past 11 years, the number of HIV cases in Indonesia peaked in 2019 (50,282 cases) [1]. The highest percentage of HIV cases was reported in the 25-year to 49-year age group (70.0%), followed by the 20-year to 24-year age group (14.9%) and the over-50-year age group (10.2%). The percentage of HIV cases was 67.6% in men and 32.4% in women, with a 2:1 men-to-women ratio [4].

One of the main reasons for the increased number of new HIV infections is a lack of knowledge about HIV/AIDS [5]. Several previous studies showed that in many settings, knowledge about HIV is still low [5–7]. In Ethiopia, only 25.2% of women had a comprehensive knowledge of HIV/AIDS (95% confidence interval [CI], 24.5 to 25.9) [5]. The corresponding rate was less than 50% in Vietnam [6] and Yemen [7]. In Indonesia, only 15% of women and 16% of married men had a comprehensive knowledge of HIV transmission and prevention [8].

Low levels of knowledge about HIV are related to higher levels of misconception about HIV transmission [9]. Misconceptions about HIV can also be influenced by area of residence, level of education, employment status [10], health literacy [11], and wealth index [9]. A misunderstanding or lack of knowledge about HIV/AIDS often contributes to fear of the disease and rejection of people living with HIV/AIDS (PLWHA) [8]. The lack of knowledge about HIV is also a perceived facilitator of stigma towards PLWHA [12]. Eight out of 10 women and married men in Indonesia discriminate against PLWHA. This discriminatory attitude towards PLWHA is most likely related to ignorance of the mechanisms of HIV transmission [8].

People living with HIV are often stigmatized, experiencing avoidance behaviors (e.g., refusal to share food, hold hands, or sit nearby), gossip and verbal abuse, and social rejection (e.g., ostracism, loss of respect and standing) [13]. Several studies reported that stigma towards PLWHA often occurs within families by parents, siblings, relatives, or in-laws [14]. They may even experience discrimination from health workers [15,16]. A lack of knowledge about HIV, fear of contracting HIV, personal values, religious beliefs, sociocultural values and norms [15], educational background, and marital status [17] are factors reported to be associated with stigma and discrimination against PLWHA. Family members encourage PLWHA to remain silent about their illness, to avoid social rejection [18]. A qualitative study in Indonesia emphasized that stigma against PLWHA causes them to hide their HIV status from relatives and the community [19]. Stigma negatively affects the social and psychological quality of life and the resilience of PLWHA [20].

In Indonesia, only 6.0% of women aged 15–49 years and 8.6% of men aged 15–49 years reported an attitude of acceptance towards PLWHA. Furthermore, only 43.9% of women and 44.6% of men said that HIV cannot be transmitted by mosquito bites; 64% of women and 54% of men would not buy fresh vegetables from a shopkeeper who had HIV; 45.8% of women said that HIV cannot be transmitted by supernatural means; and 35% of women and 29% of men think that a woman teacher who is not sick, but is infected with HIV, should not be allowed to continue teaching [8].

One of the strategies for overcoming HIV/AIDS in Indonesia is strengthening partnerships and community participation, including the private sector, the business world, and other organizations, at both the national and international level. This strategy aimed to reduce stigma and discrimination in society. An analysis of HIV and other sexually transmitted infection programs in 2015–2019 showed that no data were available to measure the main indicators of zero new HIV infections, zero AIDS-related deaths, and zero discrimination [21]. This study aimed to identify the factors related to misconceptions about HIV transmission and the stigma against PLWHA in Indonesia, with the goal of contributing to HIV/AIDS prevention programs in Indonesia.

## MATERIALS AND METHODS

### Study design

This cross-sectional study used secondary data from the 2017 Indonesia Demographic and Health Survey (IDHS). The dependent variables were misconceptions about HIV transmission and stigma against PLWHA. The independent variables were area of residence, educational level, employment status, wealth index quintile, reading ability, and access to mass media. Misconceptions about HIV transmission also served as an independent variable for the outcome of stigma against PLWHA.

### Setting

The 2017 IDHS sample covered 1,970 census blocks in urban and rural areas and was expected to obtain responses from 49,250 households. The sample frame of the 2017 IDHS was the Master Sample of census blocks from the 2010 Indonesia Population Census. The frame for the household sample selection was the updated list of ordinary households in the selected census blocks. This list did not include institutional households, such as orphanages, police/military barracks, prisons, or special households (boarding houses with a minimum of 10 people). Fieldwork took place from July 24 to September 30, 2017.

### Participants

The 3,023 participants in this study were respondents to the 2017 IDHS, who were 17–45 years of age and married. There were 1,620 women (53.6%) and 1,403 men (46.4%).

### Measurements

The socio-demographic variables that were collected included age, gender, educational status, employment status, area of residence, and wealth index quintile. Other variables were reading ability, access to mass media (newspapers/magazines, radio, or television), misconceptions about HIV transmission, and stigma against PLWHA. Misconceptions about HIV transmission were measured using 4 indicators: (1) People can get HIV from mosquito bites (yes/no/do not know); (2) People can get HIV by sharing food with a person who has HIV (yes/no/do not know); (3) People can get HIV because of witchcraft or other supernatural means (yes/no/do not know); (4) It is possible for a person who appears to be healthy to have HIV (yes/no/don’t know).

A “yes” answer to questions 1, 2, or 3 and a “no” answer to question 4 were considered to indicate misconceptions about HIV.

Stigma against PLWHA was measured using the indicators below:

(1) Would you buy fresh vegetables from a shopkeeper or vendor if you knew that this person had HIV/AIDS (yes/no/do not know)? (2) If a member of your family was infected with HIV, you would not want it to remain a secret (yes/no/do not know)? (3) If a member of your family became sick with HIV/AIDS, would you be willing to care for her or him in your own household (yes/no/do not know)? (4) A woman teacher who appears to be healthy, but is infected with HIV, should be allowed to continue teaching (yes/no/do not know).

Respondents were said to stigmatize PLWHA if they answered “no” to at least one of the indicators above.

### Statistical analysis

Descriptive statistics were calculated for all participant characteristics (Table 1). Backward multivariate analysis was conducted to determine the factors related to misconceptions about HIV transmission and stigma against PLWHA. As a result, area of residence, employment status, and access to mass media were excluded from the model for the outcome of misconceptions about HIV transmission. Area of residence was excluded from the model for the outcome of stigma against PLWHA.

### Ethics statement

Not applicable as the manuscript did not involve any experimentation.

## RESULTS

This study included a total of 3,023 respondents. The sample characteristics are summarized in Table 1.

The average age of respondents was 32.41 years (standard deviation [SD], 7.33) and most were in the age range of 26–35 years (44.3%). Almost all (98.4%) of respondents had heard of HIV/AIDS, but 75.9% of respondents showed misconceptions about HIV transmission. Surprisingly, almost all (94.7%) of respondents stigmatized PLWHA.

### Misconceptions about HIV transmission

In this study, 75.9% of respondents expressed misconceptions about HIV transmission. Educational level and wealth index quintile were significantly related to misconceptions about HIV transmission, with reading ability as a control variable. The most influential variable was educational level. When compared to respondents with post-secondary education, respondents with no education had a 5.3 times greater risk (p<0.001) of experiencing misconceptions about HIV transmission, respondents with primary education had a 4.4 times greater risk (p<0.001), and respondents with secondary education had a 2.7 times greater risk (p<0.001). When compared to the richest quintile, respondents who were in the poorest quintile had a 1.8 times higher chance of having misconceptions about HIV transmission (p<0.001), respondents in the poorer quintile had a 2.2 times higher chance (p<0.001), and respondents in the middle quintile had a 2.4 times higher chance (p<0.001). Respondents in the richer quintile had a 1.6 times higher chance of having misconceptions about HIV transmission when compared to respondents in the richest quintile (p<0.05) (Table 2).

### Stigma against PLWHA

Three variables were significantly related to stigma against PLWHA: educational level, employment status, and wealth index quintile, with reading ability and misconceptions about HIV transmission as control variables. The wealth index quintile was the variable with the greatest influence. Respondents in the richest quintile were 3.3 times (p<0.001) more likely to stigmatize PLWHA than the poorest respondents. Respondents who worked were 3.0 times (p=0.001) more likely to stigmatize PLWHA than those who did not work. Respondents with secondary education were 2.7 times (p<0.05) more likely to stigmatize PLWHA than respondents with higher education (Table 3).

## DISCUSSION

Almost all (98.4%) of respondents had heard of HIV/AIDS, but 75.9% of respondents had misconceptions about HIV transmission. More than 50% of respondents believed that HIV can be transmitted through mosquito bites, sharing food with PLWHA, and witchcraft or other supernatural means, and that it is impossible for someone who looks healthy to be infected with HIV. These findings correspond to several previous studies in various countries [5,9,10]. We found that educational level and the wealth index quintile were associated with misconceptions about HIV transmission, with people who had lower levels of education most likely to express misconceptions. This result is supported by studies done in Ethiopia [10], Malawi [9], and Bangladesh [22]. This may be because educated individuals have more access to information about HIV/AIDS than their counterparts [5]. Accurate knowledge is essential to reduce the various misconceptions about HIV [10]. These findings suggest a need for awareness-raising campaigns targeting individuals who have not had formal schooling, specifically using mass media to attract a wider audience and improve overall knowledge about HIV/AIDS. The results of this study also indicate that there are still social and cultural barriers that prevent access to comprehensive knowledge. In general, effective educational programs are needed to increase individual knowledge [14].

Individuals in the poorest, poorer, middle, and richer wealth index quintiles were more likely to report misconceptions about HIV transmission than those in the richest quintile. This result is supported by studies done in Vietnam [23] and Malawi [9]. A high socioeconomic status improves access to media sources and education, therefore increasing the likelihood of knowledge about HIV/AIDS [5].

This study reveals that the stigma against PLWHA is still widespread in Indonesia and other countries [24,25]. Having a secondary education, being currently employed, and being included in the richest category of the wealth index are variables positively related to stigma against PLWHA. People with low levels of education may have a poor understanding of HIV/AIDS (including misconceptions about how it is transmitted) leading to an increased likelihood of stigma toward people living with HIV [26]. Educated people have a greater awareness of the prognosis of PLWHA and the availability of anti-retroviral treatments [27]. This study also shows that people who are currently working tend to stigmatize PLWHA.

This study found that respondents in the richest quintile were less compassionate towards PLWHA than respondents in the poorest quintile. This finding contradicts studies conducted in Nigeria [26] and Tajikistan [28]. A possible explanation is that HIV is considered a disease of the poor. Poverty increases risky behavior towards HIV/AIDS such as transactional gender. Poverty also leads to fewer opportunities for work and education. On a broader scale, financial shortfalls can limit educational opportunities, access to health care, and access to jobs. These conditions create a favorable environment for the spread of HIV [29]. Therefore, the richest might judge PLWHA as belonging to poorer classes, thereby stigmatizing PLWHA.

The limitation of this study was a cross-sectional study and therefore could not determine causality.

This study showed that many Indonesian people still experience misconceptions about HIV transmission and stigmatize PLWHA. Educational level and the wealth index quintile were related to misconceptions about HIV transmission. Respondents with a lower educational level were more likely to experience misconceptions about HIV transmission. Respondents who were in the poorest, poorer, middle and richer wealth index quintiles were more likely to have misconceptions about HIV transmission than respondents who were in the richest wealth index quintile. Educational level, employment status, and wealth index quintile were predictors of stigma against PLWHA. Future studies should focus more on educational programs or interventions aimed at increasing public knowledge and awareness, promoting compassion towards PLWHA, and emphasizing respect for the rights of PLWHA, particularly among the poor and uneducated. By increasing public knowledge about HIV/AIDS, it is hoped that the stigma against PLWHA can be reduced.

## Figures and Tables

**Table 1:** Descriptive characteristics of the analytic sample (n=3,023)

Characteristics	n (%)
Age, mean±SD (yr)	32.41±7.33

Age (yr)	
17–25	634 (21.0)
26–35	1,340 (44.3)
36–45	1,049 (34.7)

Gender	
Men	1,403 (46.4)
Women	1,620 (53.6)

Ever heard of AIDS	
No	47 (1.6)
Yes	2,976 (98.4)

Area of residence	
Rural	2,103 (69.6)
Urban	920 (30.4)

Educational level	
No education	1,961 (64.9)
Primary	393 (13.0)
Secondary	559 (18.5)
Higher	110 (3.6)

Wealth index quintile	
Poorest	721 (23.9)
Poorer	718 (23.8)
Middle	666 (22.0)
Higher	570 (18.9)
Highest	348 (11.5)

Employment status	
No	434 (14.4)
Yes	2,589 (85.6)

Reading ability	
Unable	2,189 (72.4)
Able	834 (27.6)

Access to mass media	
No	914 (30.2)
Yes	2,109 (69.8)

Knowledge about HIV transmission	
People can get HIV from mosquito bites	
Yes	1,704 (56.4)
No	885 (29.3)
Don’t know	434 (14.4)
People can get HIV by sharing food with a person who has HIV	
Yes	1,684 (55.7)
No	955 (31.6)
Don’t know	384 (12.7)
People can get HIV because of witchcraft or other supernatural means	
Yes	1,835 (60.7)
No	471 (15.6)
Don’t know	717 (23.7)
It is possible for a healthy-looking person to have HIV	
Yes	1,840 (60.9)
No	717 (23.7)
Don’t know	466 (15.4)
Misconception about HIV transmission	
Yes	2,293 (75.9)
No	730 (24.1)

Attitude towards PLWHA	
If a member of your family was infected with HIV, you would not want it to remain a secret	
Yes	1,368 (45.3)
No (want it to remain a secret)	1,655 (54.7)
You would buy fresh vegetables from a shopkeeper or vendor if you knew that this person had HIV/AIDS	
Yes	1,153 (38.1)
No	1,798 (59.5)
Don’t know	72 (2.4)
If a member of your family became sick with HIV/AIDS, you would be willing to care for her or him in your own household	
No	2,316 (76.6)
Yes	588 (19.5)
Don’t know	119 (3.9)
A women teacher who is infected with HIV, but is not sick, should be allowed to continue teaching	
Yes	1,398 (46.2)
No	1,443 (47.7)
Don’t know	182 (6.0)
Stigma against PLWHA	
Yes	2,863 (94.7)
No	160 (5.3)

SD, standard deviation; HIV, human immunodeficiency virus; AIDS, acquired immune deficiency syndrome; PLWHA, people living with HIV/AIDS.

**Table 2:** Descriptive statistics and multivariable regression model exploring variation in misconceptions about HIV transmission

Variables	Misconceptions about HIV transmission		Total	p-value	OR (95% CI)

	No	Yes			
Area of residence					
Rural	420 (20.0)	1,683 (80.0)	2,103 (69.6)	-	-
Urban	310 (33.7)	610 (66.3)	920 (30.4)	-	-

Educational level					
No education	352 (18.0)	1,609 (82.0)	1,961 (64.9)	<0.001	5.28 (2.83, 9.84)
Primary	90 (22.9)	303 (77.1)	393 (13.0)	<0.001	4.37 (2.52, 7.58)
Secondary	214 (38.3)	345 (61.7)	559 (18.5)	<0.001	2.71 (1.73, 4.22)
Higher	74 (67.3)	36 (32.7)	110 (3.6)		1.00 (reference)

Employment status					
No	126 (29.0)	308 (71.0)	434 (14.4)	-	-
Yes	604 (23.3)	1,985 (76.9)	2,589 (85.6)	-	-

Wealth index quintile					
Poorest	152 (21.1)	569 (78.9)	721 (23.9)	<0.001	1.79 (1.32, 2.43)
Poorer	133 (18.5)	585 (81.5)	718 (23.8)	<0.001	2.23 (1.64, 3.04)
Middle	120 (18.0)	546 (82.0)	666 (22.0)	<0.001	2.38 (1.74, 3.26)
Richer	162 (28.4)	408 (71.6)	570 (18.8)	0.003	1.58 (1.17, 2.12)
Richest	163 (46.8)	185 (53.2)	348 (11.5)		1.00 (reference)

Reading ability					
Unable	401 (18.3)	1,788 (81.7)	2,189 (72.4)	-	-
Able	329 (39.4)	505 (60.6)	834 (27.6)	0.395	0.82 (0.53, 1.29)

Access to mass media					
No	190 (20.8)	724 (79.2)	914 (30.2)	-	-
Yes	540 (25.6)	1,569 (74.4)	2,109 (69.8)	-	-

Values are presented as number (%).

HIV, human immunodeficiency virus; OR, odds ratio; CI, confidence interval.

**Table 3:** Descriptive statistics and multivariable regression model exploring variation in stigma against PLWHA

Variables	Stigma against PLWHA		Total	p-value	OR (95% CI)

	Yes	No			
Area of residence					
Rural	2,013 (95.7)	90 (4.3)	2,103 (69.6)	-	-
Urban	850 (92.4)	70 (7.6)	920 (30.4)	-	-

Educational level					
No education	1,875 (95.6)	86 (4.4)	1,961 (64.9)	0.323	1.95 (0.52, 7.36)
Primary	381 (96.9)	12 (3.1)	393 (13.0)	0.787	1.17 (0.37, 3.77)
Secondary	504 (90.2)	55 (9.8)	559 (18.5)	0.019	2.73 (1.18, 6.33)
Higher	103 (93.6)	7 (6.4)	110 (3.6)	-	1.00 (reference)

Employment status					
No	423 (97.5)	11 (2.5)	434 (14.4)	-	1.00 (reference)
Yes	2,440 (94.2)	149 (5.8)	2,589 (85.6)	0.001	3.04 (1.61, 5.71)

Wealth index quintile					
Poorest	693 (96.1)	28 (3.9)	721 (23.9)	-	1.00 (reference)
Poorer	697 (97.1)	21 (2.9)	718 (23.7)	0.233	0.70 (0.39, 1.25)
Middle	631 (94.7)	35 (5.3)	666 (22.0)	0.240	1.36 (0.81, 2.27)
Richer	540 (94.7)	30 (5.3)	570 (18.9)	0.435	1.24 (0.72, 2.14)
Richest	302 (86.8)	46 (13.2)	348 (11.5)	0.000	3.34 (1.94, 5.75)

Reading ability					
Unable	2,097 (95.8)	92 (4.2)	2,189 (72.4)	-	1.00 (reference)
Able	766 (91.8)	68 (8.2)	834 (27.6)	0.744	1.19 (0.42, 3.31)

Access to mass media					
No	871 (95.3)	43 (4.7)	914 (30.2)	-	-
Yes	1,992 (94.5)	117 (5.5)	2,109 (69.8)	-	-

Misconception about HIV transmission					
Yes	2,189 (95.5)	104 (4.5)	2,293 (75.9)	-	1.00 (reference)
No	674 (92.3)	56 (7.7)	730 (24.1)	0.069	0.72 (0.50, 1.03)

Values are presented as number (%).

PLWHA, people living with HIV/AIDS; HIV, human immunodeficiency virus; OR, odds ratio; CI, confidence interval.
